# New 30-Noroleanane Triterpenoid Saponins from *Holboellia coriacea* Diels

**DOI:** 10.3390/molecules21060734

**Published:** 2016-06-04

**Authors:** Wenbing Ding, Ye Li, Guanhua Li, Hualiang He, Zhiwen Li, Youzhi Li

**Affiliations:** 1 Hunan Provincial Engineering & Technology Research Center for Biopesticide and Formulation Processing, Hunan Agricultural University, Changsha 410128, China; dingwenb119@hunau.edu.cn (W.D.); 15273361055@139.com (Y.L.); lgh202@126.com (G.L.); hhl_1234@126.com (H.H.); lizhw809718@aliyun.com (Z.L.); 2 Hunan Co-Innovation Center for Utilization of Botanical Functional Ingredients, Changsha 410128, China

**Keywords:** *Holboellia coriacea*, nortriterpenoids, saponins, cytotoxicity

## Abstract

Three new 30-noroleanane triterpenoid saponins, akebonoic acid 28-*O*-*β*-d-glucopyranosyl-(1′′→6′)-*β*-d-glucopyranosyl ester (**1**), akebonoic acid 28-*O*-(6′′-*O*-caffeoyl)-*β*-d-glucopyranosyl-(1′′→6′)-*β*-d-glucopyranosyl ester (Holboelliside A, **2**) and 3*β*,20*α*,24-trihydroxy-29-norolean-12-en-28-oic acid 3-*O*-(6′-*O*-caffeoyl)-*β*-d-glucopyranoside (Holboelliside B, **3**) were isolated from the stems of *Holboellia coriacea* Diels, together with five known compounds, eupteleasaponin VIII (**4**), 3*α*-akebonoic acid (**5**), quinatic acid (**6**), 3*β*-hydroxy-30-norhederagenin (**7**) and quinatoside A (**8**). The structures of these compounds were determined on the basis of spectral and chemical evidence. Compounds **1**–**5** were evaluated for their inhibitory activity against three human tumors HepG2, HCT116 and SGC-7901 cell lines *in vitro*.

## 1. Introduction

*Holboellia coriacea* Diels belonging to family Lardizabalaceae, is an evergreen woody vines mainly distributed in Qinling Mountain region of China at altitudes of 500–2000 m [1]. The fruit of *H. coriacea*, edible berries, can be used to wine, while the stems and roots of the plant have been used as Chinese folk medicine for treating arthritis and rheumatism paralysis [1,2]. To date there are very few reports about the chemical constituents of this species. Recently, a phytochemical study revealed six triterpenoid saponins, including two noroleanane-type ones from a methanol extract of root of *H. coriacea* [2]*.* Noroleanane triterpenoids are recognized as characteristic constituents of the family Lardizabalaceae, some of which were revealed to show significant bioactivities [3,4,5]. Our interest in the chemistry of noroleanane triterpenoids prompted us to continue the phytochemical study of *H. coriacea*, whereby three new 30-noroleanane triterpenoid saponins (**1**–**3**) and five known ones (**4**–**8**) were obtained from the stem of the plant. Herein, we report the isolation, structure elucidation, and cytotoxic activity of these compounds.

## 2. Results and Discussion

**Figure 1:**
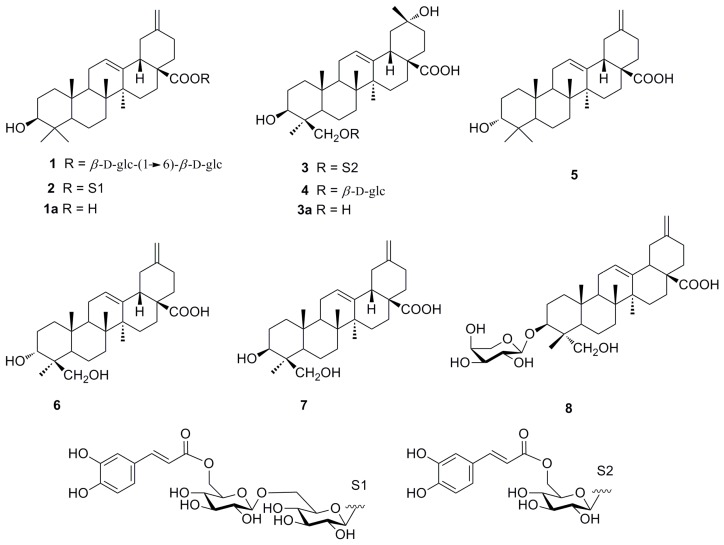
Structures of compounds **1**–**8** isolated from *H. coriacea*, as well as structures of **1a** and **3a** mentioned in the text.

The dried stems of *H. coriacea* were extracted using 95% aq. EtOH. The EtOH extract residue was suspended in water and then partitioned successively with petroleum ether and EtOAc. Column chromatography of the EtOAc-soluble fraction yielded three new triterpenoid saponins (**1**–**3**), which were identified by NMR techniques and HRESIMS, and five known compounds **4**–**8** that were identified as by comparison of their NMR and MS data with those reported in the literature as eupteleasaponin VIII (**4**) [6], 3α-akebonoic acid (**5**) [7], quinatic acid (**6**) [8], 3*β*-hydroxy-30-norhederagenin (**7**) [9] and quinatoside A (**8**) [10] (Figure 1).

### 2.1. Identification of New Compounds

Compound **1** was obtained as white solid powder (MeOH). The positive HR-ESIMS of **1** showed an [M + Na]^+^ at *m/z* 787.4248, which, taken together with the ^13^C-NMR data analysis, indicated the molecular formula C_41_H_64_O_13_. The ^13^C-NMR spectrum displayed 41 signals, of which 12 carbons were assignable to sugar moieties and 29 carbons to the aglycone moiety (Table 1). On the basis of ^13^C-NMR and DEPT spectra, the carbons for the aglycone were identified as five methyls, four olefinic carbons, an oxy-methine carbon, a carbonyl carbon, 10 methylenes, three methines, and five quaternary carbons. The HSQC spectrum of **1** displayed corresponding five angular methyl groups (δ_H_ 0.94 (s, 3H); 1.02 (s, 3H); 1.12 (s, 3H); 1.20 (s, 3H); 1.22 (s, 3H)), a broad singlet olefinic proton at δ_H_ 5.45, and two exo-methylene protons at δ_H_ 4.65 (s) and 4.71 (s). These NMR data suggested that the structure of **1** should be a noroleanane triterpenoid saponin [4]. After comparison of the ^1^H and ^13^C-NMR data with those of closely related analogues, the aglycone was characterized as 3*β*-hydroxy-30-norolean-12,20(29)-dien-28-oic acid (akebonoic acid) [7], a common triterpene aglycon occurring in the genera of Lardizabalaceae. The *β*-orientation of the hydroxyl group at C-3 was confirmed by an NOE correlation of H-3 with Hα-5 (Figure S7) and the proton spin-coupling constant of H-3 (δ_H_ 3.44 (dd, *J* = 9.9, 5.6 Hz)). In addition, the ^1^H and ^13^C-NMR spectra exhibited two anomeric proton signals at δ_H_ 6.21 (d, *J* = 8.0 Hz) and 5.00 (d, *J* = 7.7 Hz), as well as twelve carbon signals at δ_C_ 95.2 (glc-1′), 73.3 (glc-2′), 78.1 (glc-3′), 70.4 (glc-4′), 77.3 (glc-5′), 69.0 (glc-6′), 104.8 (glc-1′′), 74.6 (glc-2′′), 77.8 (glc-3′′), 71.0 (glc-4′′), 77.9 (glc-5′′), and 62.1 (glc-6′′), which correspond with that of published data for a sugar moiety of *β*-d-glucopyranosyl (1′′→6′) *β*-d-glucopyranosyl [11]. Furthermore, the sugar moiety was found to be attached to C-28 via an ester linkage from correlations between H-1′ (δ_H_ 6.21) and carboxyl carborn (δ_C_ 175.3) observed in the HMBC spectrum (Figure S6). Based upon all of the above evidence, the structure of **1** was elucidated as akebonoic acid 28-*O*-*β*-d-glucopyranosyl-(1′′→6′)-*β*-d-glucopyranosyl ester.

Compound **2** gave quasi-molecular ions at *m*/*z* 949 [M + Na]^+^ and 961 [M + Cl]^−^ by ESI-MS. The molecule formula of C_50_H_70_O_16_ was confirmed by HR-ESIMS. By comparison of the ^1^H and ^13^C-NMR data (Table 2) with those of **1**, compound **2** was found to have the same aglycone (akebonoic acid) and sugar moiety as those in **1**, but an additional *trans*-caffeoyl moiety which was proposed according to the presence of an aromatic ABX system (δ_H_ 7.54, br s; 7.09, dd, *J* = 8.2, 1.9 Hz; 7.17, d, *J* = 8.2 Hz), together with two coupled doublets (*J* = 15.8 Hz) at δ_H_ 6.61 and 7.91 and the signal of an ester carbon C=O at δ_C_ 167.1 [12]. The *trans*-caffeoyl moiety was deduced to be bound at C-6′′ of the sugar moiety via an ester linkage from the deshielded signal of the protons H-6′′ (Table 2) and from long-range correlations of H-6′′ (4.88 (dd, *J* = 11.8, 5.5 Hz)/5.03 (dd, *J* = 11.8, 1.6 Hz)) with C=O (δ_C_ 167.1) observed in the HMBC spectrum (Figure S13). The aforementioned structure was confirmed by alkaline and acid hydrolysis of **2**, which yielded akebonoic acid (**1a**) and d-glucose. Therefore, compound **2** was determined as akebonoic acid 28-*O*-(6′′-*O*-caffeoyl)-*β*-d-glucopyranosyl-(1′′→6′)-*β*-d-glucopyranosyl ester, a new caffeoylation of nortriterpenoid saponin that we have named Holboelliside A.

Compound **3** was obtained as a light yellow powder. The molecular formula was shown to be C_44_H_62_O_13_ by its HR-ESIMS and ^13^C-NMR data. Its ^1^H- and ^13^C-NMR spectra (Table 3) were similar to those of compound **4**, eupteleasaponin VIII [6], except for the presence of a typical ABX aromatic spin system at δ_H_ 7.49 (1H, d, 1.6 Hz), 7.18 (1H, d, 8.2 Hz), and 7.05 (1H, dd, 8.2, 1.6 Hz), as well as two coupled doublets (*J* = 15.8 Hz) protons for a *trans*-caffeoyl unit [12]. The deshielded signal of the protons H-6′ (4.95 (dd, *J* = 11.4, 4.7 Hz)/5.03 (br d, *J* = 10.1 Hz)) of the glucose moiety and the correlation between H-6′ and C=O (δ_C_ 167.4) in the HMBC spectrum indicated that the *trans*-caffeoyl moiety is attached to C-6′ via an ester linkage. Moreover, In the NOESY spectrum (Figure S21), H*α*-5 was correlated with H-3 and H_3_-23, and H*β*-18 was correlated with H_3_-29, confirming the stereostructures of the aglycone should be 3*β*,20*α*,24-trihydroxy-29-norolean-12-en-28-oic acid [13]. Consequently, the structure of **3** was determined to be 3*β*,20*α*,24-trihydroxy-29-norolean-12-en-28-oic acid 3-*O*-(6′-*O*-caffeoyl)-*β*-d-glucopyranoside, a cafeoyl ester of **4** that we have named Holboelliside B.

It is noteworthy that the caffeoylation of nortriterpenoid saponins (**2** and **3**) were isolated from family Lardizabalaceae for the first time. This kind of esterification is particularly important for saponins for increasing their solubility in water (and hence to favor its lymphatic transport), thus the esterification of many secondary metabolites generally represents one of the last steps of their biosynthesis [12]. Because noroleanane triterpenoids are recognized as characteristic constituents of the family Lardizabalaceae [3], the occurrence of those caffeoylation derivatives in *Holboellia coriacea* may have a role as chemotaxonomic markers for *Holboellia*, which is worthy of further study.

### 2.2. Cytotoxicity Assay

The cytotoxicity of compounds **1**–**5** against three human tumors HepG2, HCT116 and SGC-7901 cell lines were assessed, using a sulforhodamine B (SRB) method. The resulting IC_50_ values are displayed in Table 4. Only compound **5** showed interesting cytotoxicity against HCT-116, HepG2 and SGC-7901 cell lines, with IC_50_ values of 9.1, 15.2 and 41.0 μM, respectively. Previous cytotoxicity studies on this type of nortriterpenoids suggested that the exocyclic double bond at C-20(29) might be an important active center to “maintain” their potential cytotoxicity [5], while the present result suggested that the saccharide unit attached at C-28 could greatly reduce the *in vitro* cytotoxicity.

## 3. Materials and Methods

### 3.1. General Experimental Procedures

Optical rotations were determined using a Perkin-Elmer 341 polarimeter (PerkinElmer Co., Waltham, MA, USA). Ultraviolet (UV) spectra were taken on an UVmini-1240 spectrometer (Shimadzu Co., Kyoto, Japan) and HRESIMS spectra on an API QSTAR mass spectrometer (Applied Biosystem/MSD Sciex, Concord, ON, Canada). The ^1^H, ^13^C, and 2D NMR spectra were recorded on a Bruker Avance-600 or a Bruker DRX-400 instrument using TMS as an internal standard. Column chromatography was performed on silica gel 60 (200–300 mesh, Qingdao Marine Chemical Ltd., Qingdao, China). For Preparative TLC plates (HSGF254, Jiangyou silicone Development Co., Ltd., Yantai, China), Sephadex LH-20 (GE Healthcare, Uppsala, Sweden) and Develosil ODS (50 μm, Nomura Chemical Co. Ltd., Osaka, Japan) were used.

### 3.2. Plant Material

The stems of *H. coriacea* were collected from Xiangxi, Hunan Province, China, in December 2012, and identified by Prof. Zhang Dai-gui (Key Laboratory of Plant Resources Conservation and Utilization, Jishou University, Jishou, China). A voucher specimen (zdg-20121203) has been deposited at the Hunan Agricultural University.

### 3.3. Extraction and Isolation

Dried stems of *H. coriacea* (5 kg) were powdered and extracted with 95% EtOH (3 × 15 L, 48 h each) at room temperature, then evaporated *in vacuo* to give 550 mg of residue. The residue was further suspended in H_2_O (1 L) and sequentially extracted with petroleum ether (PE, 3 × 1 L) and EtOAc (3 × 1 L), to yield a PE-soluble fraction (15.4 g) and an EtOAc-soluble fraction (61.8 g). The EtOAc-soluble fraction was subjected to silica gel column chromatography (CC) (100–200 mesh) with elution of CHCl_3_-MeOH (100:0→60:40, *v*/*v*) to give nine fractions (Fr. A1–A9). Fraction A2 was decolorized on MCI gel column eluting with a gradient solvent system consisting of 20%, 85% and 100% (MeOH-H_2_O, *v*/*v*). The fraction eluted with 85% MeOH was chromatographed on MPLC (ODS-C_18_) using MeOH-H_2_O (50:50→100:0, *v*/*v*) system, to produce seven sub-fractions (A2-a–A2-g). Compound **6** (28 mg) was obtained from sub-fraction A2-e which was purified by normal silica gel CC (petroleum ether-EtOAc, 9:1), compound **7** (16 mg) from sub-fraction A2-f was purified on a Sephadex LH-20 column (MeOH), and compound **5** (30 mg) was recrystallized from sub-fraction A2-e. Similarly, Fractions A6 (6.0 g), A7 (6.0 g) and A8 (8.3 g) were fractionated by an ODS-C_18_ column with elution of MeOH-H_2_O (50:50→100:0, *v*/*v*) system, respectively. Compound **8** (30 mg) precipitated from sub-fraction A6-f and compound **4** (30 mg) was purified from sub-fraction A7-d by gel filtration on a Sephadex LH-20 column (MeOH). Whereas, sub-fraction A8-d and A8-f were purified successively on preparative TLC (GF254) plates using chloroform-methanol-water (10:3:1, *v*/*v*/*v*) as mobile solvent and Sephadex LH-20 column using MeOH as eluent, to obtain **3** (10 mg), **1** (22 mg), and **2** (19 mg).

### 3.4. Spectroscopic Data of ***1**–**3***

Compound **1**: White solid powder, ${\left[\alpha \right]}_{\text{D}}^{25}$ + 125.0 (*c* = 0.5, MeOH); IR (KBr) υ_max_ 3415, 2930, 1741, 1679, 1433, 1385, 1133, 1047 cm^−1^; ^1^H-NMR (400 MHz, pyridine-*d_5_*) and ^13^C-NMR (100 MHz, pyridine-*d_5_*) spectroscopic data, see Table 1; positive ion ESIMS *m*/*z*: 787 [M + Na]^+^; negative ESIMS *m*/*z*: 763 [M − H]^−^, 799 [M + Cl]^−^; HR-ESIMS *m*/*z*: 787.4248 [M + Na]^+^ (calcd for C_41_H_64_O_13_ Na, 787.4239).

Compound **2**: light yellow powder, ${\left[\alpha \right]}_{\text{D}}^{25}$ + 25.7 (*c* = 2.3, MeOH); UV (MeOH) λ _max_ nm (log ε) 345 (4.00), 242 (2.32), 129 (2.94); IR (KBr) υ_max_ 3435, 3420, 3414, 2933, 2854, 1745, 1689, 1620, 1451, 1435, 1380, 1045 cm^−1^; ^1^H-NMR (600 MHz, pyridine-*d_5_*) and ^13^C-NMR (125 MHz, pyridine-*d_5_*) spectroscopic data, see Table 1; positive ion ESIMS *m*/*z*: 949 [M + Na]^+^; negative ESIMS *m*/*z*: 961 [M + Cl]^−^; HR-ESIMS *m*/*z*: 961.4378 [M + Cl]^−^ (calcd for C_50_H_70_O_16_Cl, 961.4358).

Compound **3**: light yellow powder, ${\left[\alpha \right]}_{\text{D}}^{25}$ + 45.0 (*c* = 2.0, MeOH); UV (MeOH) λ _max_ nm (log ε) 329 (2.59), 244 (1.78), 217 (2.41); IR (KBr) υ_max_ 3435, 3069, 1719, 1648, 1621, 1298, 1073 cm^−1^; ^1^H-NMR (600 MHz, pyridine-*d_5_*) and ^13^C-NMR (125 MHz, pyridine-*d_5_*) spectroscopic data, see Table 1; positive ion ESIMS *m*/*z*: 821 [M + Na]^+^; negative ESIMS *m*/*z*: 797 [M − H]^−^, 833 [M + Cl]^−^; HR-ESIMS *m*/*z*: 797.4109 [M − H]^−^ (calcd for C_44_H_61_O_13_, 797.4118).

### 3.5. Chemical Hydrolysis of ***1**–**3***

Alkaline and Acid Hydrolysis of **1** and **2**: A solution of saponins (**1** or **2**, 10 mg each) in 5% KOH (3 mL) was stirred at 85 °C for 4 h. The reaction mixture was acidified to pH 4.0 with 10% HCl and extracted with CHCl_3_ (3 × 10 mL). The CHCl_3_ layer was dried (anhydr. MgSO_4_) and concentrated under reduced pressure. The residue was subjected to Sephadex LH-20 CC using MeOH to afford akebonoic acid (**1a**) for the aglycone (5 mg from **1**, 3.5 mg from **2**). **1a** was identified on the basis of ^1^H and ^13^C-NMR spectrum comparisons with that of published data for akebonoic acid [7]. The aqueous layer was further concentrated and the residue in 5%–10% H_2_SO_4_ (2 drops) was heated in a boiling H_2_O bath for 1 h. The solution was extracted with *n*-BuOH (3 × 20 mL). The *n*-BuOH solution was concentrated *in vacuo* and desalted (Sephadex LH-20, MeOH) to afford the sugar residue (1.0 mg). The sugar residue was derivatized with Sigma Sil-A for 35 min at 70 °C and analyzed by GC-MS with only one peak detected (Rt = 12.19 min, EI-MS *m*/*z*: M^+^ 540) [14], which was identified as trimethylsilyl derivative of d-glucose.

Alkaline and Acid Hydrolysis of **3**: Compounds **3** (8.0 mg) in 1 N HCl (5 mL, 1,4-dioxane-H_2_O, 1:1) was heated under reflux for 8 h. After removal of the solvent, the residue was partitioned between CHCl_3_ and H_2_O. The CHCl_3_-soluble portion was evaporated and subjected to ODS column chromatography using 90% MeOH to yield 4 mg of the aglycone (**3a**) which was identified as 3*β*,20*α*,24-trihydroxy-29-norolean-12-en-28-oic acid [13]. By the method described in **1** and **2**, the sugar unit of **3** was identified as d-glucose.

Compound **1a**: ^1^H-NMR (600 MHz, pyridine-*d_5_*) δ 5.51 (1H, br t, H-12), 4.80/4.76 (2H, each s, H_2_-29, 3.60 (1H, dd, *J* = 9.6, 5.8 Hz, H-3), 3.25 (1H, dd, *J* = 13.0, 4.9 Hz, H-18), 1.25 (6H, s, H_3_-23/ H_3_-24), 1.03 (3H, s, H_3_-27), 1.01 (3H, s, H_3_-26), 0.88 (3H, s, H_3_-25); ^13^C-NMR (150 MHz, Pyr) δ 39.3 (C-1), 28.4 (C-2), 78.4 (C-3), 39.7 (C-4), 56.1 (C-5), 19.1 (C-6), 33.6 (C-7), 40.1 (C-8), 48.4(C-9), 37.7 (C-10), 24.2 (C-11), 123.3 (C-12), 144.5 (C-13), 42.4 (C-14), 28.6 (C-15), 24.1 (C-16), 47.4 (C-17), 48.3 (C-18), 42.4 (C-19), 149.5 (C-20), 38.7 (C-21), 30.8 (C-22), 29.1 (C-23), 16.9 (C-24), 15.9 (C-25), 17.7 (C-26), 26.5 (C-27), 179.9 (C-28), 107.4 (C-29).

Compound **3a**: ^1^H-NMR (600 MHz, pyridine-*d_5_*) δ 5.57 (1H, brs, H-12), 4.50 (1H, d, *J* = 10.8 Hz, H-23), 3.65/3.64(2H, m, H-3/H-23), 3.38 (1H, dd, *J* = 13.6, 5.5Hz, H-18), 1.60 (3H, s, H_3_-29), 1.56 (3H, s, H_3_-24),1.26 (3H, s, H_3_-27), 0.99 (3H, s, H_3_-26), 0.86 (3H, s, H_3_-25); ^13^C-NMR (150 MHz, Pyr) δ 38.7 (C-1), 28.3 (C-2), 80.2 (C-3), 43.2 (C-4), 56.4 (C-5), 19.1 (C-6), 33.6 (C-7), 39.8 (C-8), 48.1 (C-9), 37.1 (C-10), 23.9 (C-11), 122.6 (C-12), 144.4 (C-13), 42.1 (C-14), 28.4 (C-15), 28.4 (C-16), 46.8 (C-17), 44.4 (C-18), 48.2 (C-19), 69.9 (C-20), 36.3 (C-21), 35.2 (C-22), 23.6 (C-23), 64.5 (C-24), 16.0 (C-25), 17.0 (C-26), 26.0 (C-27), 180.7 (C-28), 25.7 (C-29).

### 3.6. Bioassay

The cytotoxicity of compounds **1**–**5** against three human cancer cell lines, human gastric carcinoma (SGC-7901), human colon carcinoma (HCT 116) and human liver hepatocellular carcinoma (HepG2) were assayed at the National Center for Drug Screening, Shanghai, China. Sulforhodamine B (SRB) (Sigma-Aldrich Chemie GmbH, Munich, Germany) was used to test the effects of the compounds on cell growth and viability [15]. Adriamycin was used as the positive control. All tests were performed in triplicate, and results are expressed as IC_50_ values.

## 4. Conclusions

The present work reported three new nortriterpenoid saponins, akebonoic acid 28-*O*-*β*-d-glucopyranosyl-(1′′→6′)-*β*-d-glucopyranosyl ester (**1**), Holboelliside A (**2**) and Holboelliside B (**3**) isolated from the stem of *Holboellia coriacea*, along with five known compounds. It is noteworthy that the two caffeoylation of nortriterpenoidal saponins (**2** and **3**) were isolated from family Lardizabalaceae for the first time. 3*α*-akebonoic acid (**5**) showed interesting cytotoxicity against HCT-116, HepG2 and SGC-7901 cell lines, with IC_50_ values of 9.1, 15.2 and 41.0 μM, respectively. In addition, the saccharide unit attached at C-28 of this type of nortriterpenoids may greatly reduce the *in vitro* cytotoxicity.

## Figures and Tables

**Table 1:** NMR spectroscopic data for compound **1** in pyridine-*d_5_*.

No.	δc	δ_H_ (mult., *J* in Hz)	No.	δc	δ_H_ (mult., *J* in Hz)
Ag-1	38.5 CH_2_	0.98 (m); 1.52 (m)	23	28.2 CH_3_	1.22 (s)
2	27.5 CH_2_	1.79–1.82 (m)	24	16.0 CH_3_	1.02 (s)
3	77.6 CH	3.44 (dd, 9.9, 5.6)	25	15.1 CH_3_	0.94 (s)
4	38.8 qC		26	17.0 CH_3_	1.12 (s)
5	55.3 CH	0.84 (d, 11.2)	27	25.5 CH_3_	1.20 (s)
6	18.3 CH_2_	1.36 (m); 1.51 (m)	28	175.3 qC	
7	32.6 CH_2_	1.35 (m); 1.47 (m)	29	106.7 CH_2_	4.65 (s); 4.71 (s)
8	39.4 qC				
9	47.6 CH	1.63 (dd, 9.7, 7.4)	Glc-1′	95.2 CH	6.21 (d, 8.0)
10	36.8 qC		2′	73.3 CH	4.12 (m)
11	23.2 CH_2_	1.90 (m)	3′	78.1 CH	4.19 (m)
12	122.9 CH	5.45 (br s)	4′	70.4 CH	4.33 (m)
13	143.0 qC		5′	77.3 CH	4.07 (m)
14	41.5 qC		6′	69.0 CH_2_	4.35 (dd, 12.1, 4.9)
15	27.7 CH_2_	1.18 (m); 2.34 (m)			4.69 (br d, 12.1)
16	23.0 CH_2_	2.06 (m); 2.14 (m)	Glc-1′′	104.8 CH	5.00 (d, 7.7)
17	46.8 qC		2′′	74.6 CH	4.00 (t, 7.7)
18	47.0 CH	3.13 (dd, 4.7, 13.4)	3′′	77.8 CH	4.21 (m)
19	41.2 CH_2_	2.20 (m); 2.59 (t, 13.4)	4′′	71.0 CH	4.19 (m)
20	147.9 qC		5′′	77.9 CH	3.88 (m)
21	29.5 CH_2_	2.09 (m); 2.20 (m)	6′′	62.1 CH_2_	4.48 (br d, 10.5)
22	37.1 CH_2_	1.74 (m); 2.03 (m)			4.32 (m)

δ in ppm; ^1^H-NMR: 400 MHz and ^13^C-NMR 100 MHz.

**Table 2:** NMR spectroscopic data for compound **2** in pyridine-*d*_5_.

No.	δc	δ_H_ (mult., *J* in Hz)	No.	δc	δ_H_ (mult., *J* in Hz)
Ag-1	38.4 CH_2_	0.96 (m); 1.51 (m)	27	25.5 CH_3_	1.17 (s)
2	27.5 CH_2_	1.79 (m); 1.82 (m)	28	175.2 qC	
3	77.6 CH	3.42 (dd, 11.0, 4.9)	29	106.8 CH_2_	4.63 (s); 4.68 (s)
4	38.8 qC		Glc-1′	95.2 CH	6.21 (d, 8.2)
5	55.3 CH	0.82 (d, 11.9)	2′	73.3 CH	4.09 (m)
6	18.2 CH_2_	1.36 (m); 1.51 (m)	3′	78.1 CH	4.18 (t, 9.0)
7	32.6 CH_2_	1.33 (m); 1.45 (m)	4′	70.4 CH	4.31 (t, 9.0 )
8	39.4 qC		5′	77.3 CH	4.05 (m)
9	47.5 CH	1.60 (dd, 10.9, 6.9)	6′	69.0 CH_2_	4.36 (dd, 11.4, 4.8)
10	36.8 qC				4.73 (br d, 11.4)
11	23.2 CH_2_	1.86 (m); 1.89 (m)	Glc-1′′	104.7 CH	5.02 (d, 7.6)
12	122.9 CH	5.40 (t, 3.6)	2′′	74.9 CH	4.02 (m)
13	142.9 qC		3′′	78.1 CH	4.18 (t, 9.0)
14	41.5 qC		4′′	70.8 CH	4.11 (m)
15	27.7 CH_2_	2.31 (m)	5′′	74.5 CH	4.01 (m)
16	23.0 CH_2_	2.02 (m); 2.12 (m)	6′′	64.0 CH_2_	4.88 (dd, 11.8, 5.5)
17	46.8 qC				5.03 (dd, 11.8, 1.6)
18	47.0 CH	3.10 (dd, 13.4, 5.1)	Caff-1′′′	126.4 qC	
19	41.1 CH_2_	2.15 (m); 2.55 (t, 13.4)	2′′′	115.3 CH	7.54 (br s)
20	147.8 qC		3′′′	147.0 qC	
21	29.5 CH_2_	2.08 (m); 2.17 (m)	4′′′	149.8 qC	
22	37.1 CH_2_	1.75 (m); 2.03 (m)	5′′′	116.1 CH	7.17 (d, 8.2 )
23	28.2 CH_3_	1.20 (s)	6′′′	121.6 CH	7.09 (dd, 8.2, 1.9)
24	16.0 CH_3_	1.01 (s)	7′′′	145.4 CH	7.91 (d, 15.8)
25	15.1 CH_3_	0.91 (s)	8′′′	114.4 CH	6.61 (d, 15.8)
26	17.0 CH_3_	1.09 (s)	9′′′	167.1 qC	

δ in ppm; ^1^H-NMR: 600 MHz and ^13^C-NMR 150 MHz.

**Table 3:** NMR spectroscopic data for compound **3** in pyridine-*d*_5_.

No.	δc	δ_H_ (mult., *J* in Hz)	No.	δc	δ_H_ (mult., *J* in Hz)
Ag-1	38.9 CH_2_	0.92 (m); 1.45 (m)	24	73.1 CH_2_	4.25 (d, 10.0); 4.36 (d, 10.0)
2	28.3 CH_2_	1.86 (m); 1.93 (m)	25	15.6 CH_3_	0.84 (s)
3	79.3 CH	3.50 (dd, 4.1, 11.7)	26	17.0 CH_3_	0.91 (s)
4	43.0 qC		27	25.8 CH_3_	1.17 (s)
5	56.5 CH	0.88 (br d, 11.7)	28	180.1 qC	
6	19.2 CH_2_	1.49 (m); 1.63 (m)	29	25.5 CH_3_	1.55 (s)
7	33.3 CH_2_	1.19 (m); 1.35 (m)			
8	39.5 qC		Glc-1′	105.6 CH	4.93 (d, 7.7)
9	47.9 CH	1.56 (m)	2′	74.9 CH	4.02 (dd, 8.3, 7.7)
10	37.0 qC		3′	78.2 CH	4.21 (dd, 8.4, 8.3)
11	23.7 CH_2_	1.82 (m)	4′	71.2 CH	4.11 (dd, 9.6, 8.4)
12	122.3 CH	5.49 (br s)	5′	75.3 CH	4.10 (m)
13	144.2 qC		6′	64.3 CH_2_	4.95 (dd, 11.4, 4.7)
14	41.8 qC				5.03 (br d, 10.1)
15	28.1 CH_2_	1.11 (m); 2.12 (m)	Caff-1′′	126.6 qC	
16	23.7 CH_2_	1.99 (m); 2.20 (m)	2′′	115.7 CH	7.49 (d, 1.6)
17	46.6 qC		3′′	147.5 qC	
18	44.2 CH	3.32 (dd, 14.3, 3.9)	4′′	150.0 qC	
19	47.9 CH_2_	1.88 (m); 2.40 (t, 13.5)	5′′	116.6 CH	7.18 (d, 8.2)
20	69.7 qC		6′′	121.9 CH	7.05 (dd, 8.2, 1.6)
21	36.0 CH_2_	1.08 (m); 2.02 (m)	7′′	145.8 CH	7.88 (d, 15.8)
22	34.9 CH_2_	2.03 (m)	8′′	114.6 CH	6.54 (d, 15.8)
23	23.3 CH_3_	1.51 (s, 3H)	9′′	167.4 qC	

δ in ppm; ^1^H-NMR: 600 MHz and ^13^C-NMR 150 MHz.

**Table 4:** Cytotoxicity against Cancer Cell Lines of compounds **1**–**5** (IC_50_, µM).

Compounds	HepG2	HCT-116	SGC-7901
1	>100	>100	87.0
2	>100	>100	>100
3	>100	>100	>100
4	>100	>100	>100
5	15.2	9.1	41.0
Adriamycin	0.46	3.3	5.1

## Supplementary Materials

The HR-ESI-MS, ^1^H- and ^13^C-NMR and 2D-NMR spectra (Figures S1–S21) of compounds **1**–**3** are available online at www.mdpi.com/1420-3049/21/6/734/s1.
