# Supplementary material for: New 30-Noroleanane Triterpenoid Saponins from Holboellia coriacea Diels
Source: Molecules. 2016 Jun 4;21(6):734. doi: 10.3390/molecules21060734 (PMC6274467; doi:10.3390/molecules21060734)
Supplement: Supplementary file 1 [file molecules-21-00734-s001.pdf]

Supplementary Materials: New 30-Noroleanane Triterpenoid Saponins from *Holboellia coriacea* Diels

Wenbing Ding, Ye Li, Guanhua Li, Hualiang He, Zhiwen Li and Youzhi Li

Acquisition Parameter

Source Type	ESI	Ion Polarity	Positive	Set Nebulizer	0.4 Bar
Focus	Active	Set Capillary	4500 V	Set Dry Heater	180 °C
Scan Begin	100 m/z	Set End Plate Offset	-500 V	Set Dry Gas	4.0 l/min
Scan End	2000 m/z	Set Collision Cell RF	800.0 Vpp	Set Divert Valve	Waste

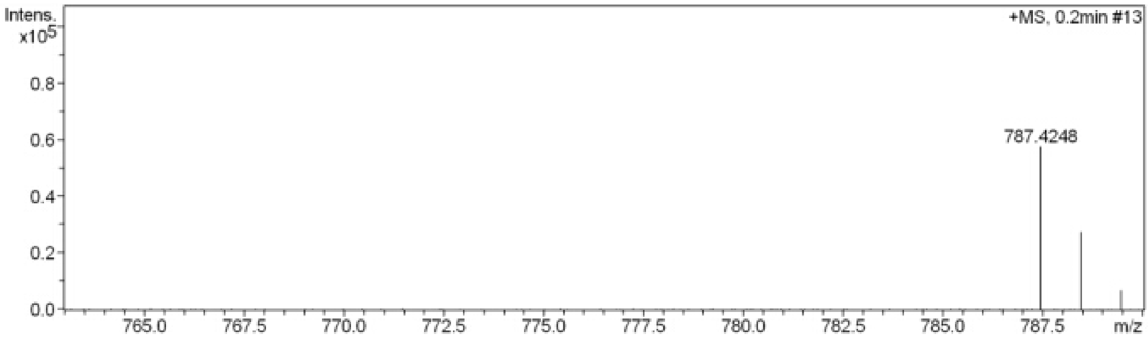

Meas. m/z	#	Formula	Score	m/z	err [ppm]	err [mDa]	mSigma	rdb	e⁻ Conf	N-Rule
787.4248	1	C 41 H 64 Na O 13	100.00	787.4239	-1.2	-0.9	11.5	9.5	even	ok

Figure S1. HR-ESIMS of compound **1**.

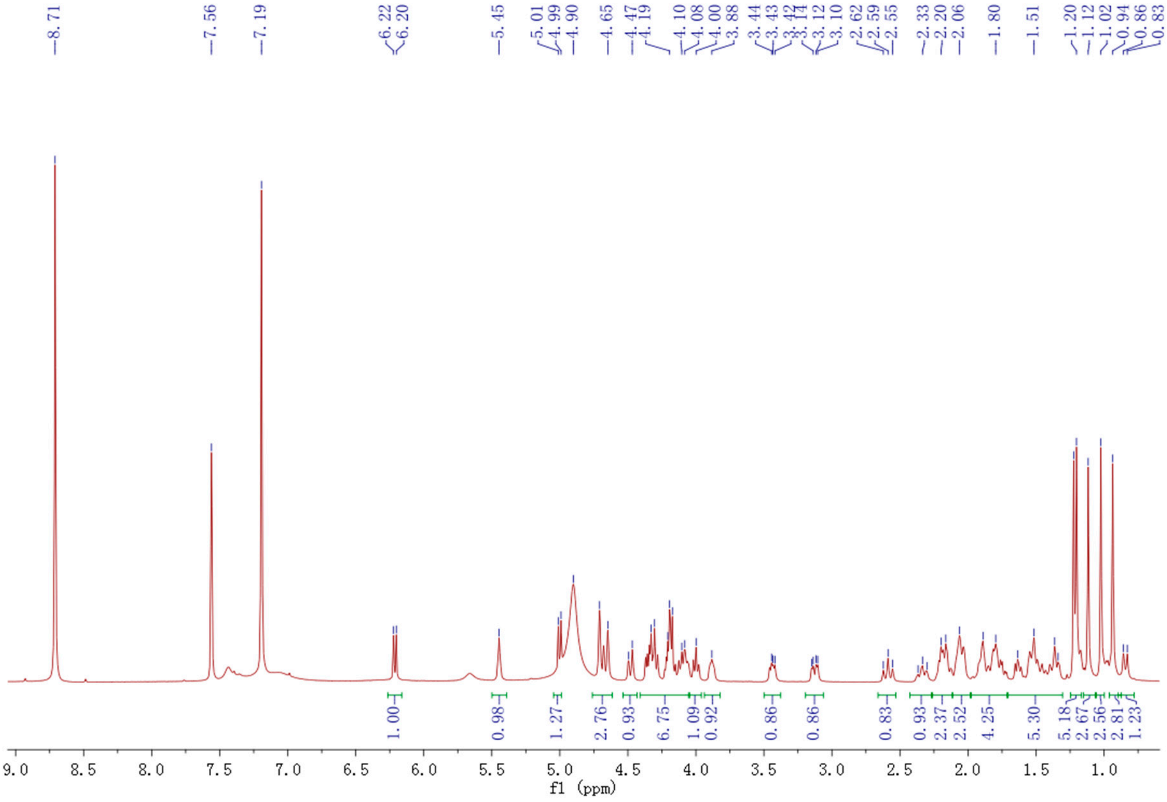

Figure S2. ^{1}H-NMR of compound **1**.

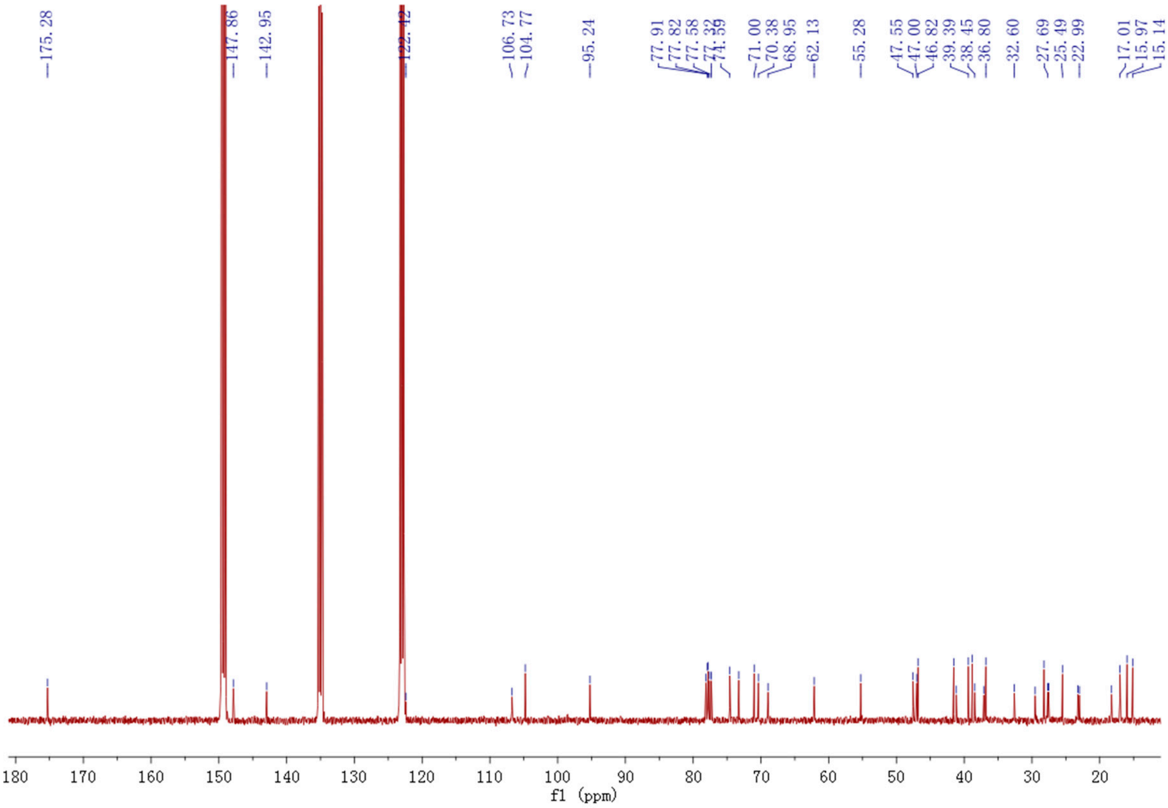

Figure S3. ^{13}C-NMR of compound **1**.

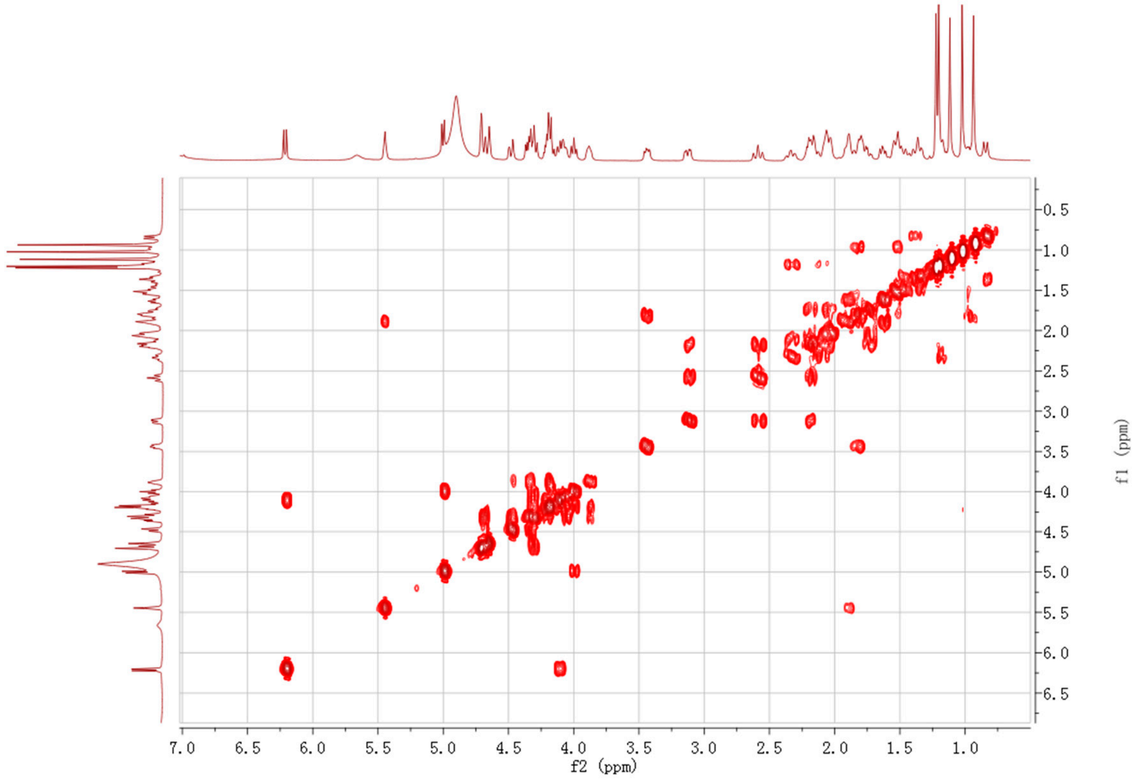

Figure S4. ^{1}H-^{1}H COSY of compound **1**.

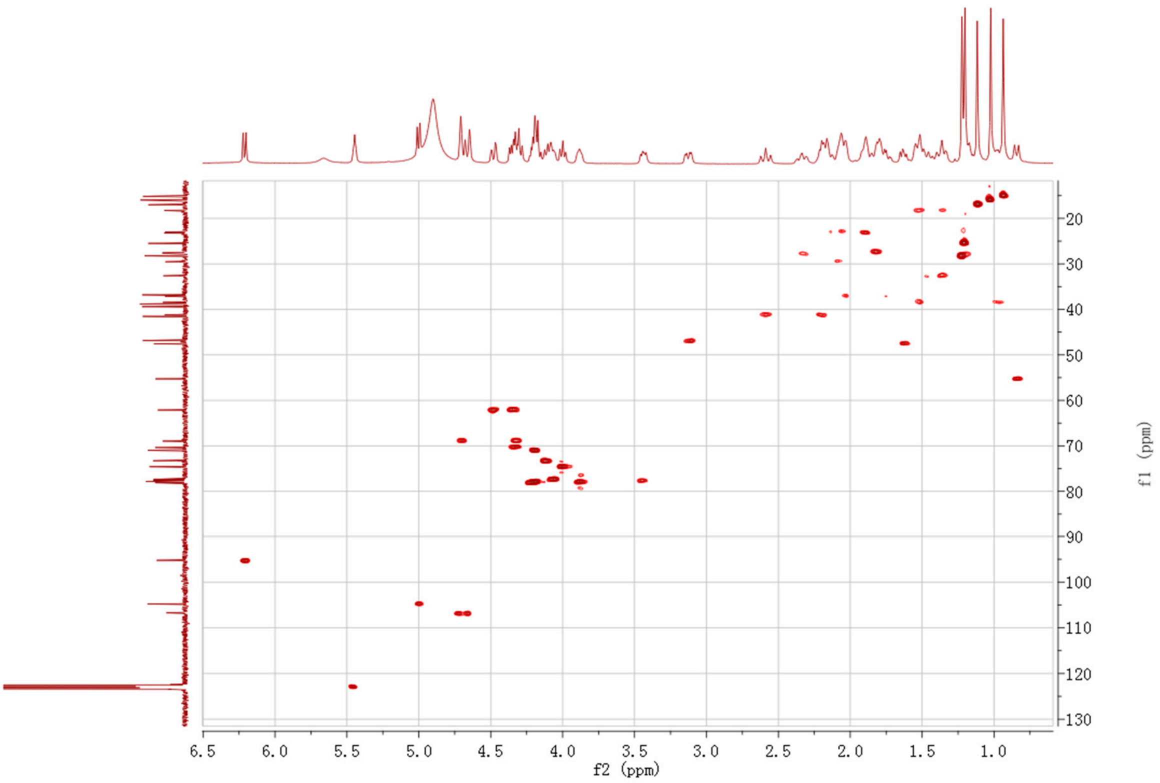

Figure S5. HSQC of compound **1**.

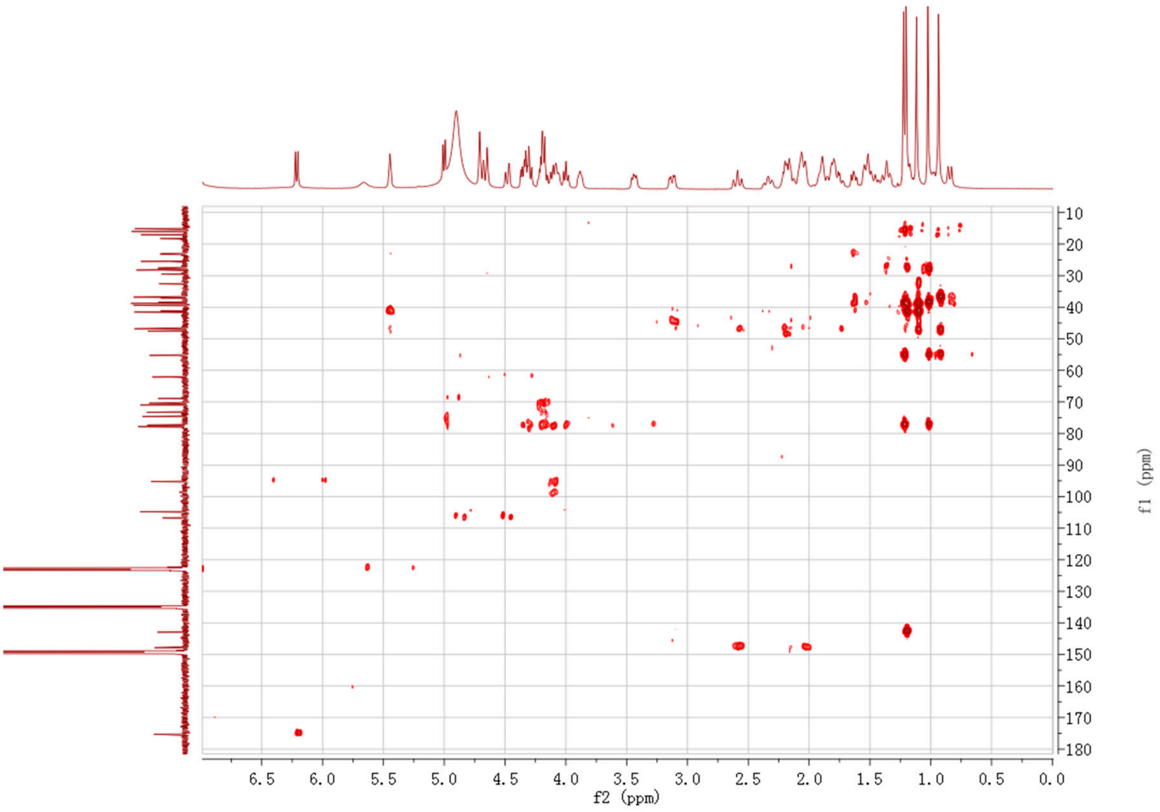

Figure S6. HMBC of compound **1**.

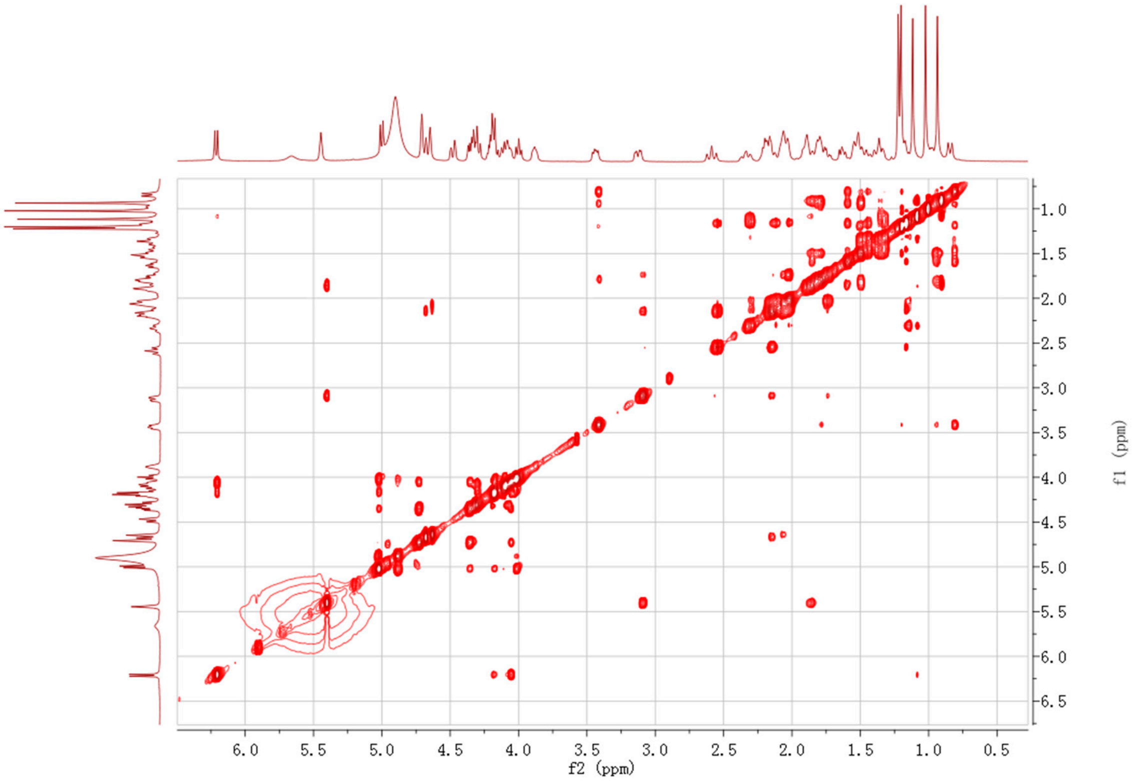

Figure S7. NOESY of compound **1**.

Acquisition Parameter

Source Type	ESI	Ion Polarity	Negative	Set Nebulizer	0.4 Bar
Focus	Active	Set Capillary	3800 V	Set Dry Heater	180 °C
Scan Begin	100 m/z	Set End Plate Offset	-500 V	Set Dry Gas	4.0 l/min
Scan End	2000 m/z	Set Collision Cell RF	550.0 Vpp	Set Divert Valve	Waste

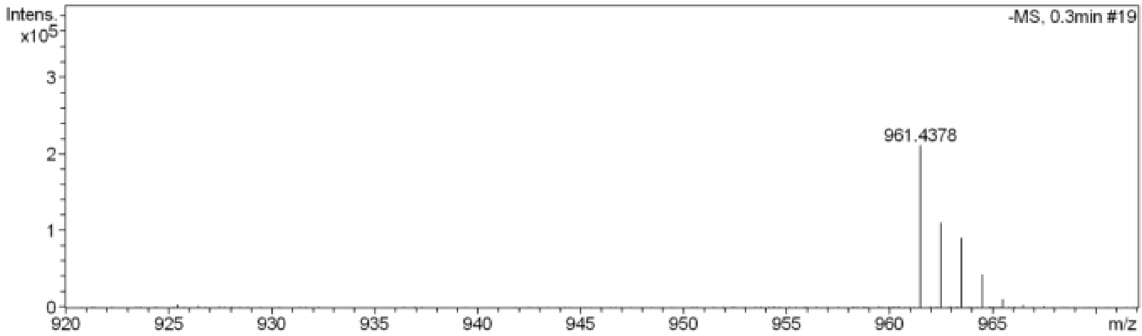

Meas. m/z	#	Formula	Score	m/z	err [ppm]	err [mDa]	mSigma	rdb	e⁻ Conf	N-Rule
925.4610	1	C 50 H 69 O 16	100.00	925.4591	-2.0	-1.9	36.6	16.5	even	ok
961.4378	1	C 50 H 70 Cl O 16	100.00	961.4358	-2.1	-2.0	35.3	15.5	even	ok

Figure S8. HR-ESIMS of compound **2**.

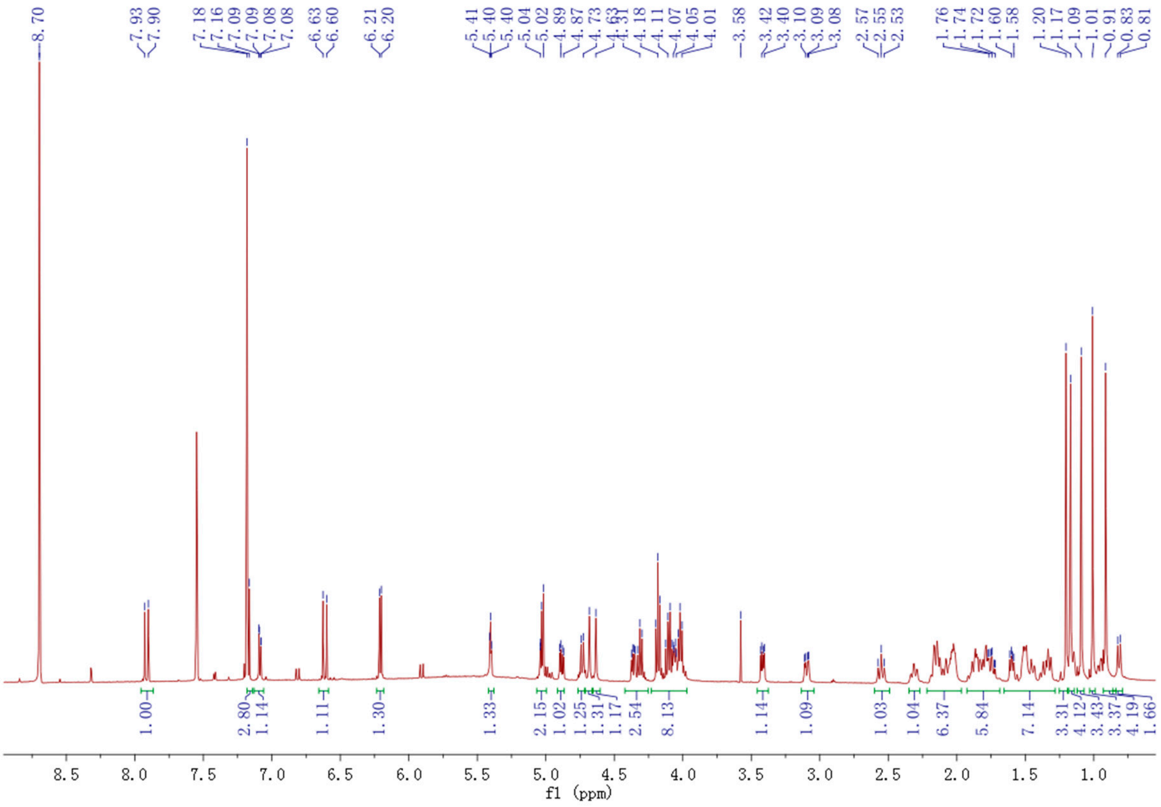

Figure S9. ^{1}H-NMR of compound **2**.

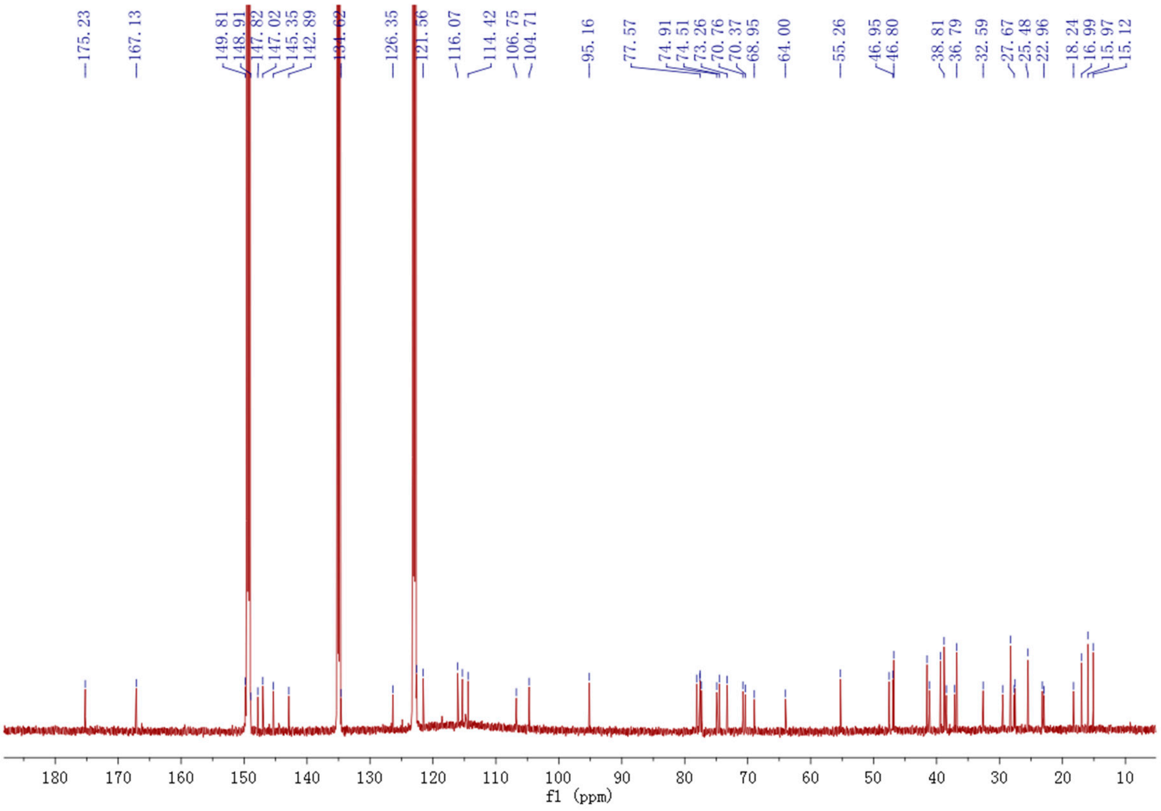

Figure S10. ^{13}C-NMR of compound **2**.

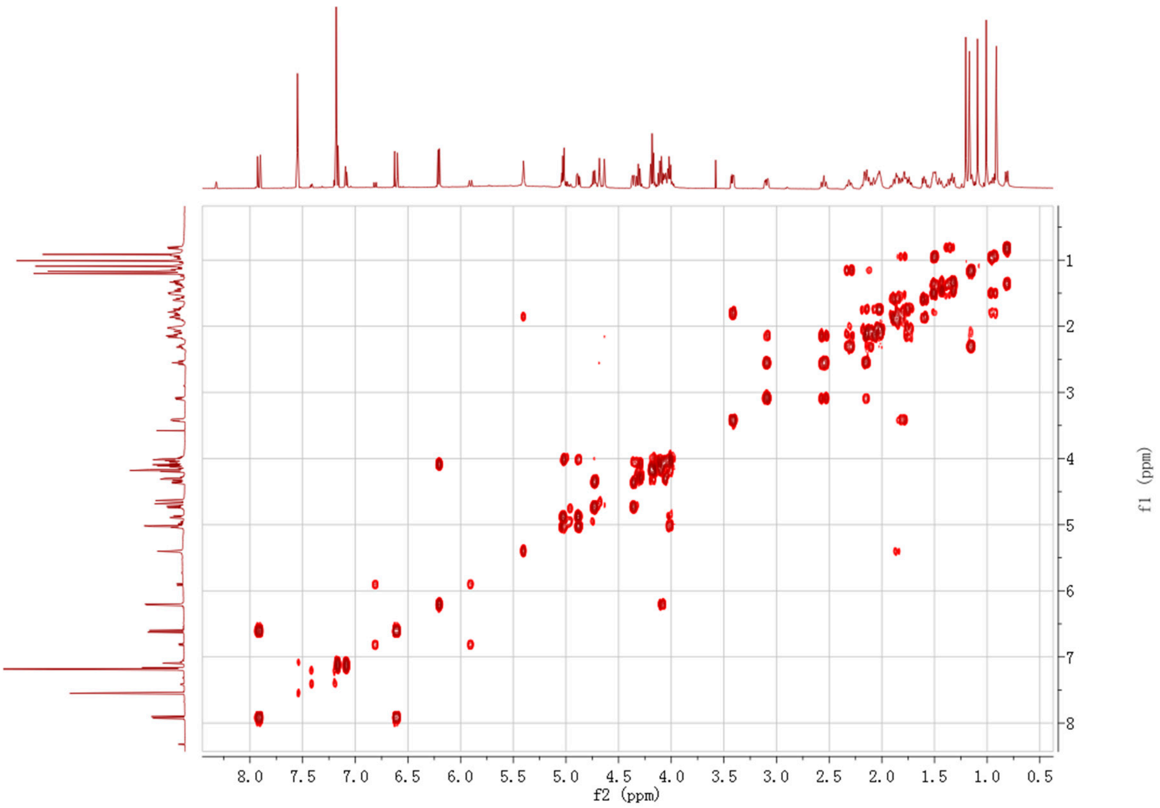

Figure S11. ^1H-^1H COSY of compound **2**.

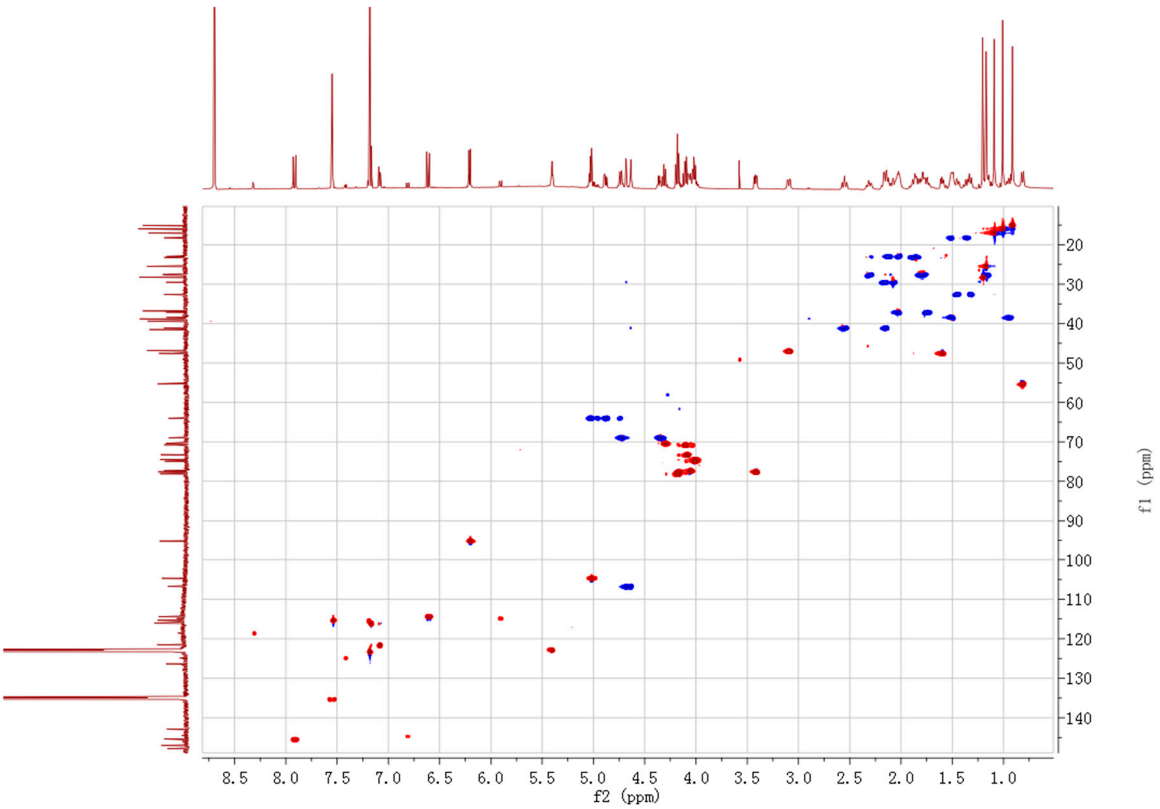

Figure S12. HSQC of compound **2**.

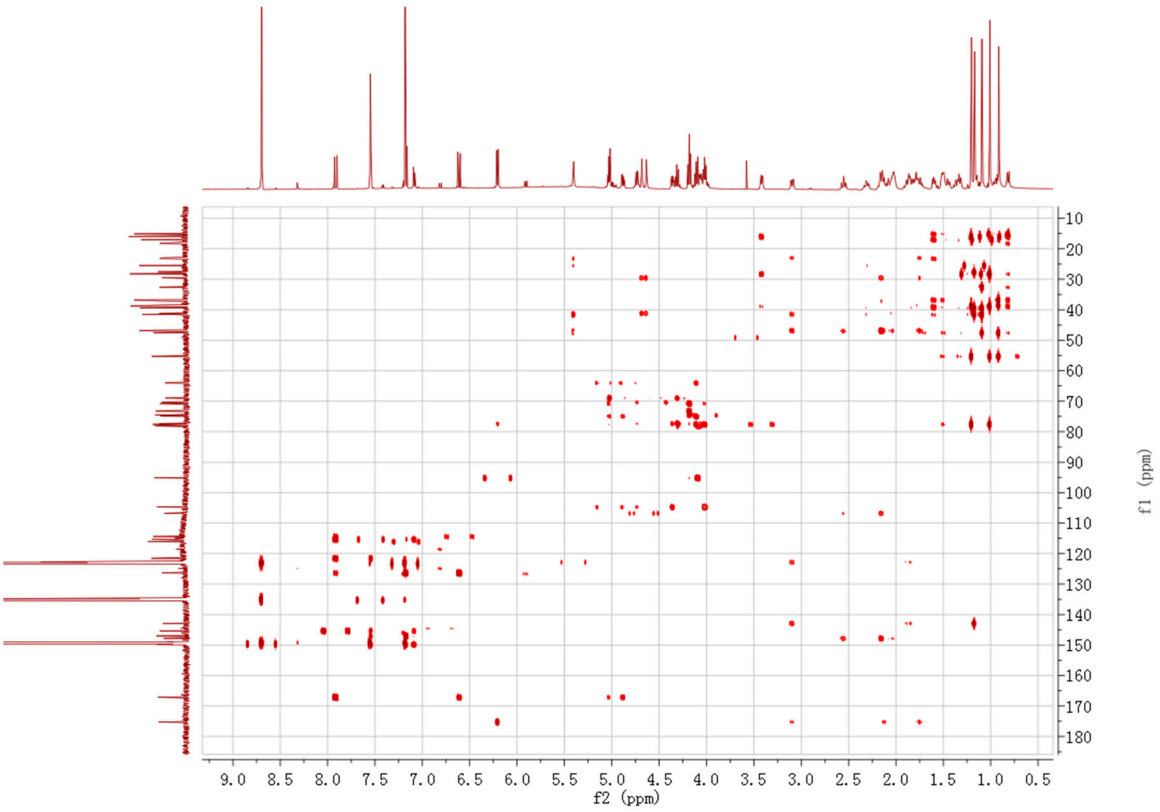

Figure S13. HMBC of compound **2**.

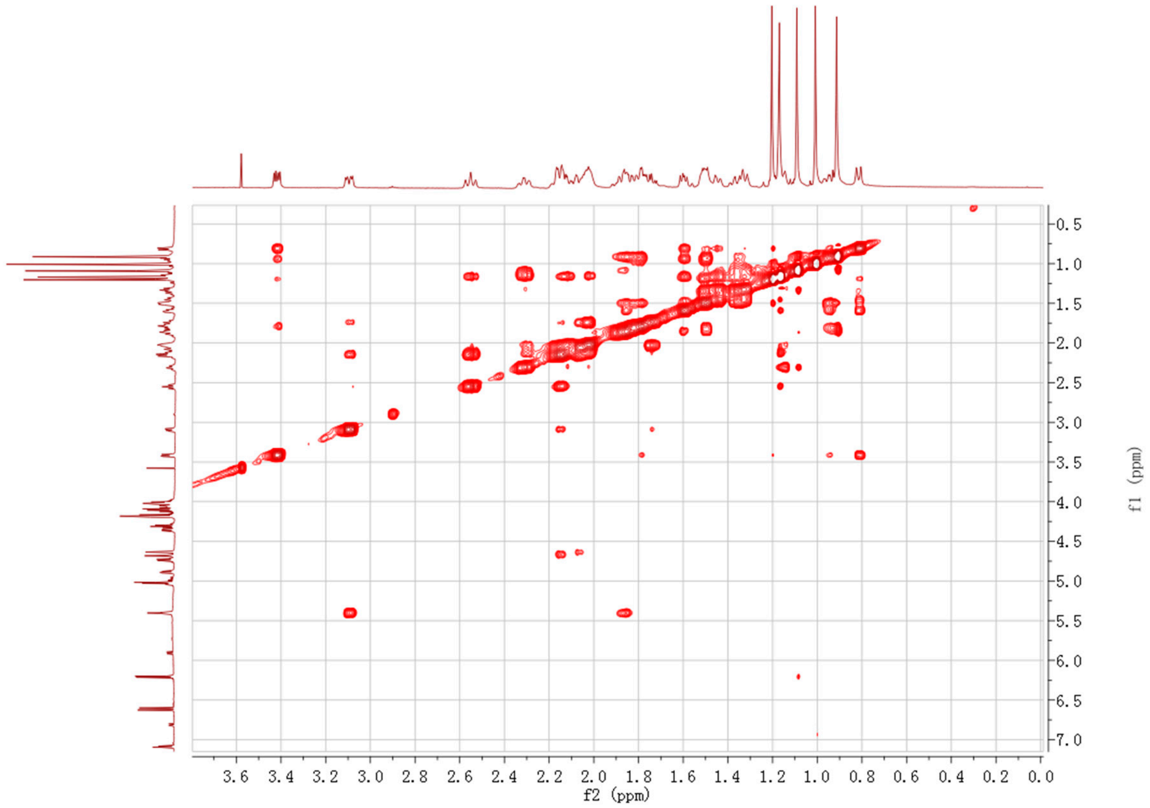

Figure S14. NOESY of compound **2**.

Acquisition Parameter

Source Type	ESI	Ion Polarity	Negative	Set Nebulizer	0.4 Bar
Focus	Active	Set Capillary	3800 V	Set Dry Heater	180 °C
Scan Begin	100 m/z	Set End Plate Offset	-500 V	Set Dry Gas	4.0 l/min
Scan End	2000 m/z	Set Collision Cell RF	550.0 Vpp	Set Divert Valve	Waste

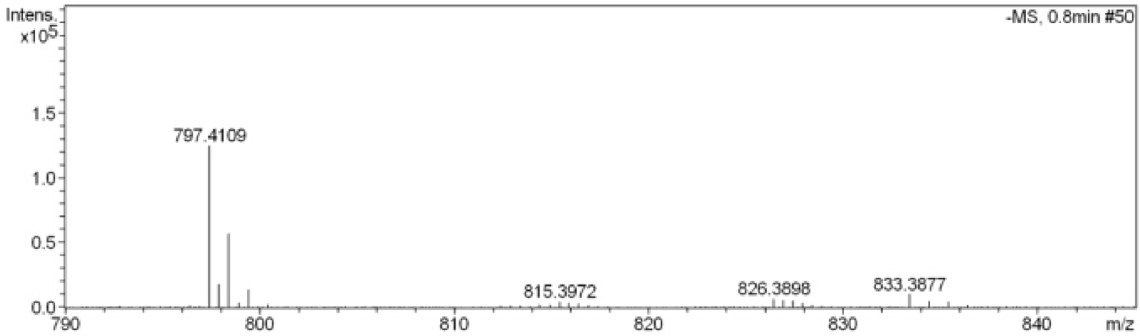

Meas. m/z	#	Formula	Score	m/z	err [ppm]	err [mDa]	mSigma	rdb	e⁻ Conf	N-Rule
797.4109	1	C 44 H 61 O 13	100.00	797.4118	1.1	0.8	25.2	14.5	even	ok

Figure S15. HR-ESIMS of compound **3**.

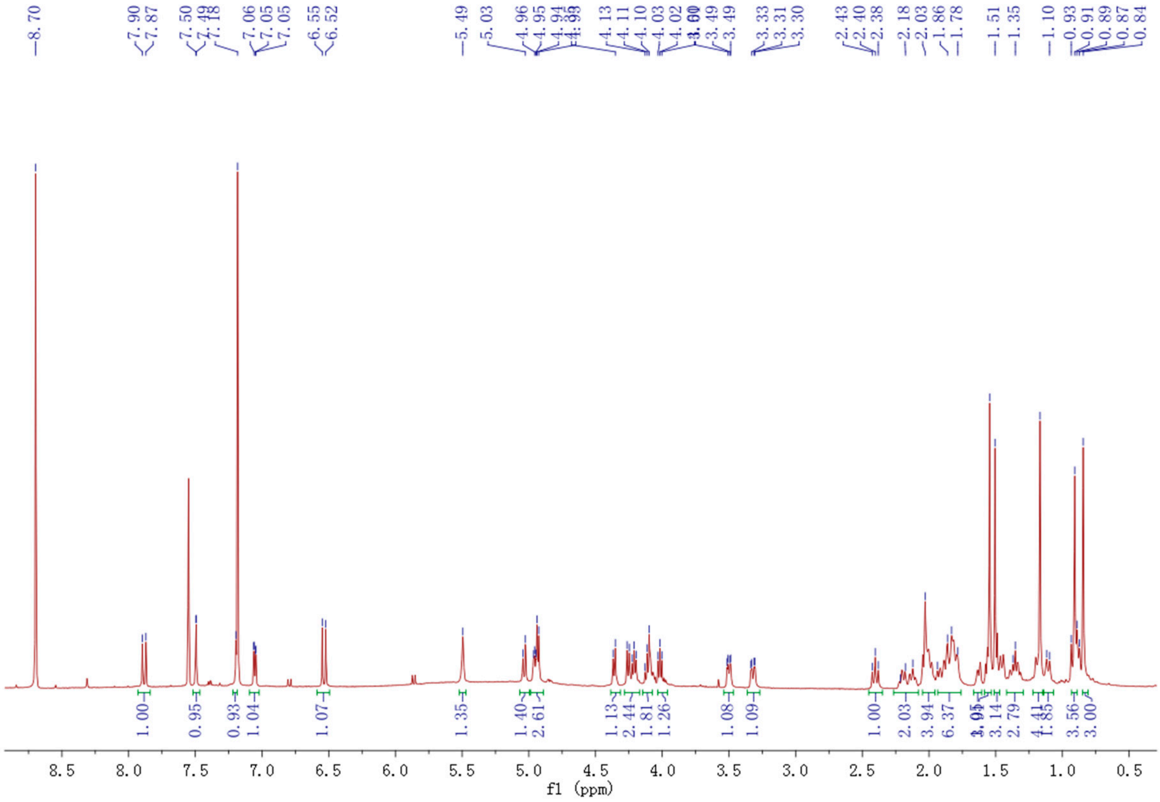

Figure S16. ^{1}H-NMR of compound **3**.

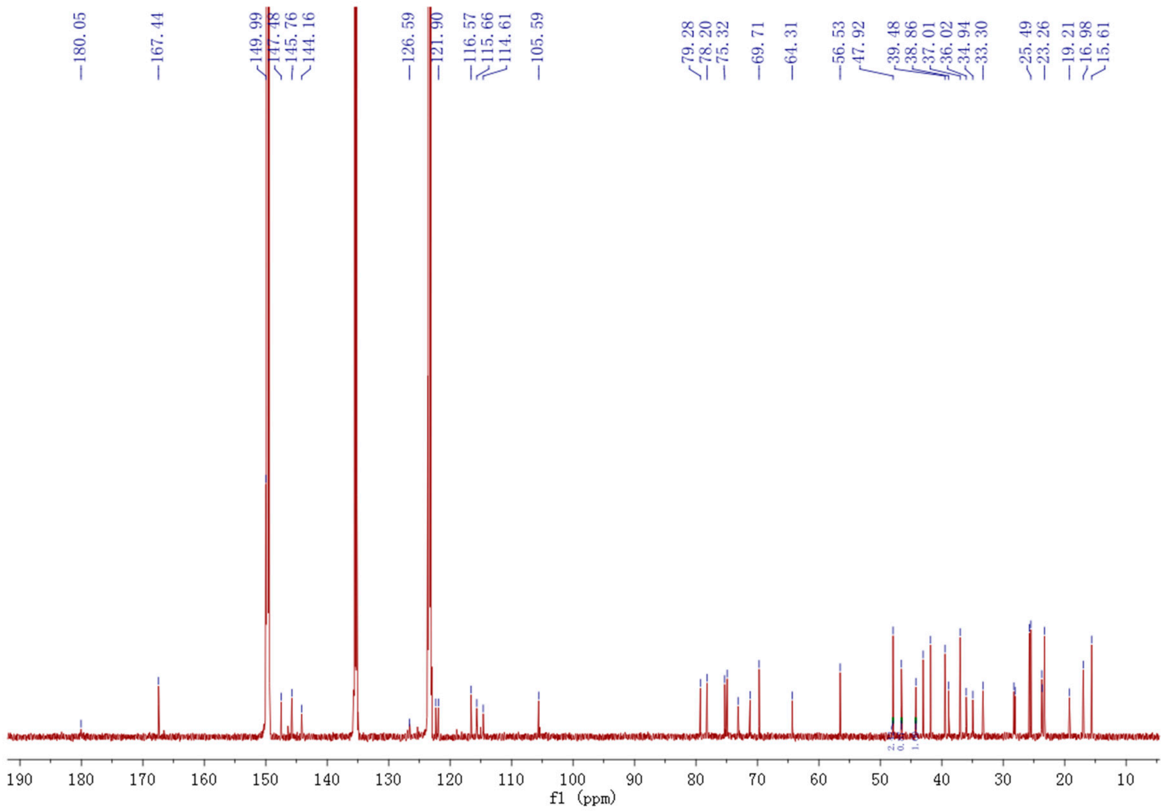

Figure S17. ^{13}C-NMR of compound **3**.

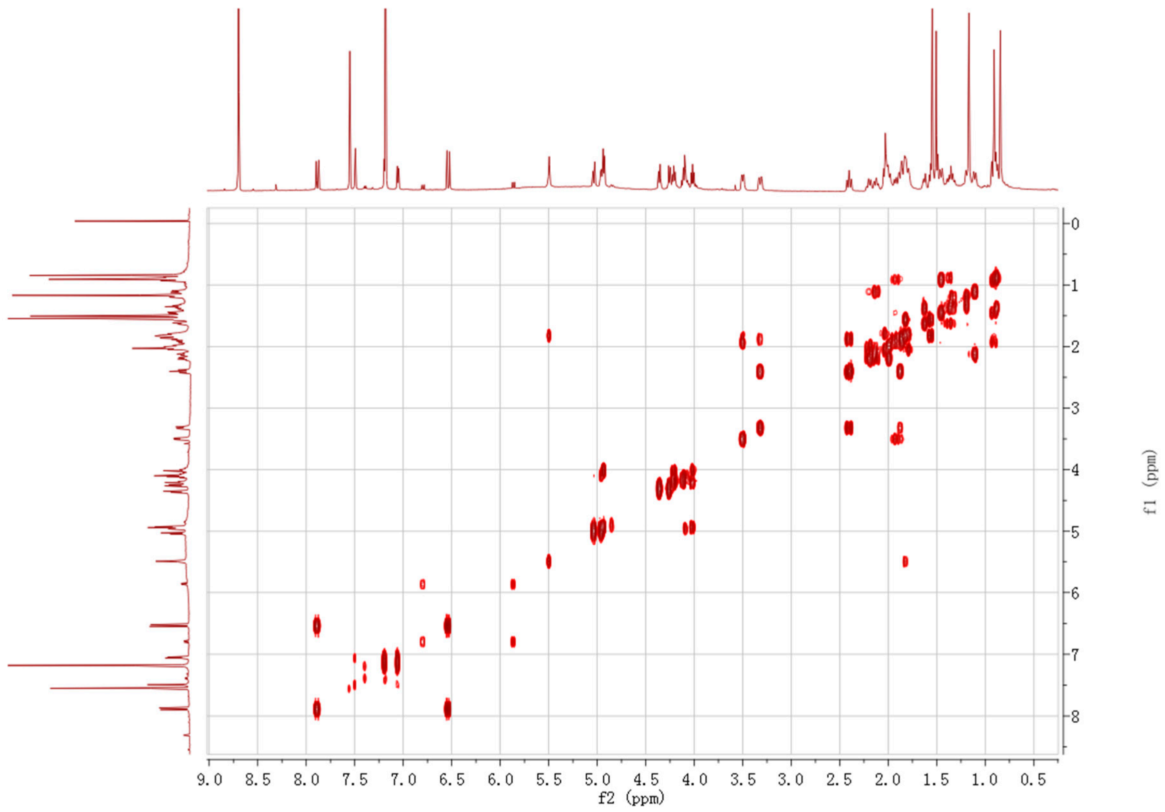

Figure S18. ^{1}H-^{1}H COSY of compound **3**.

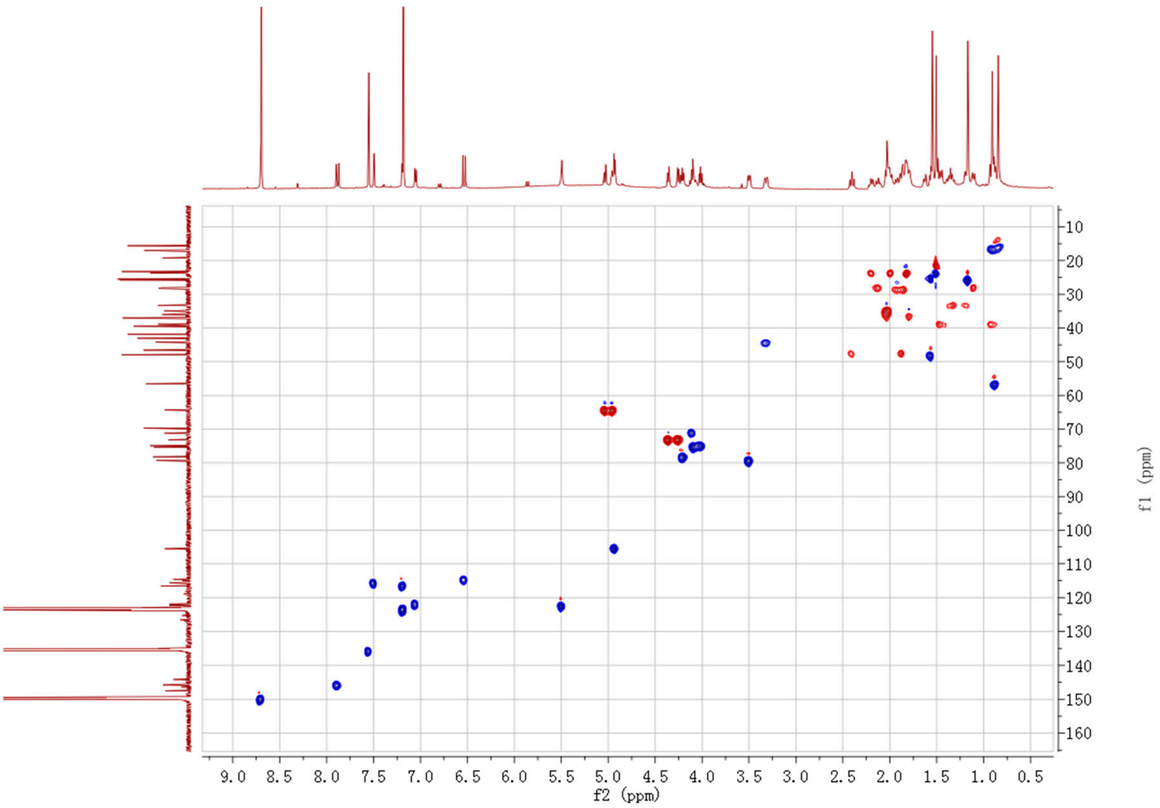

Figure S19. HSQC of compound **3**.

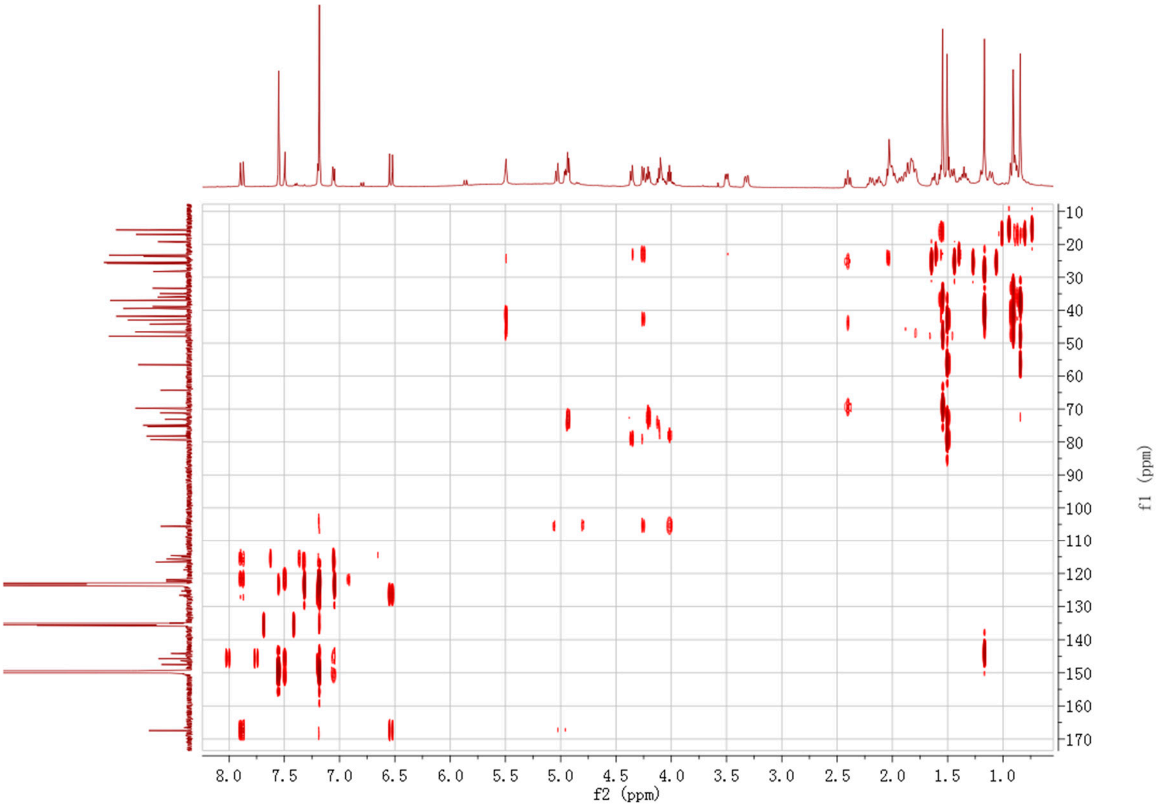

Figure S20. HMBC of compound **3**.

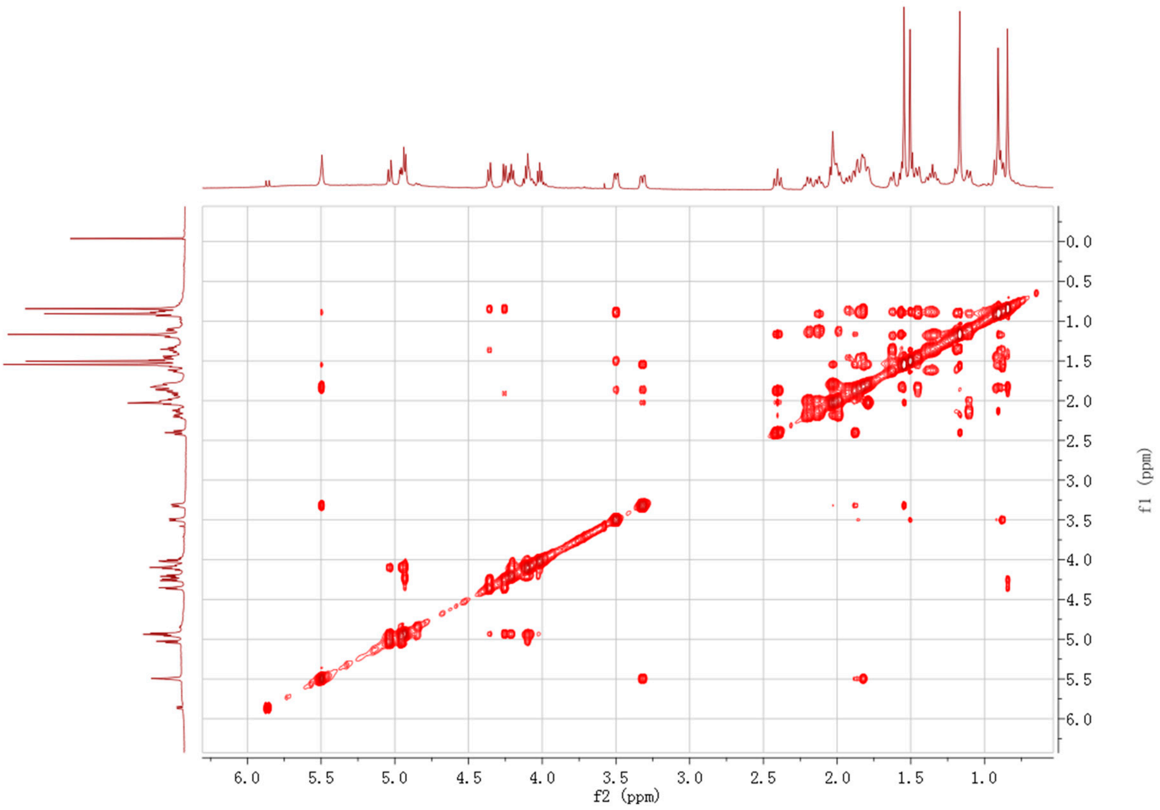

Figure S21. NOESY of compound **3**.
